# Interaction of early metabolizable protein supplementation and virginiamycin on feedlot growth performance and carcass characteristics of calf-fed Holstein steers

**DOI:** 10.1093/tas/txab228

**Published:** 2021-12-11

**Authors:** Pedro H V Carvalho, Brooke C Latack, Ruben Flores, Martin F Montano, Richard A Zinn

**Affiliations:** 1 Department of Animal Science, University of California, Holtville, CA 92250, USA; 2 Cooperative Extension, Division of Agriculture and Natural Resources, University of California, Holtville, CA 92250, USA; 3 Instituto de Investigaciones en Ciencias Veterinarias, Universidad Autónoma de Baja California, Mexicali, BC 21386, México

**Keywords:** feedlot, Holstein, performance, protein, virginiamycin

## Abstract

One hundred sixty-eight Holstein steer calves (133.4 ± 7.9 kg) were used to evaluate the influence of virginiamycin (VM) supplementation on cattle growth performance and liver abscess incidence, and the effect of feeding 100% vs. 87% of metabolizable protein (MP) requirements during the initial 112 d on growth performance, efficiency of energy utilization, and carcass characteristics. Steers were balanced by weight and assigned to 28 pens (6 steers/pen). During the initial 112-d feeding period, dietary treatments consisted of two levels of MP (100% vs. 87% of expected requirements) supplemented with or without 22.5 mg/kg VM in a 2 × 2 factorial arrangement. There were no VM × MP supplementation interactions (*P* ≥ 0.14) on any of the parameters measured in both experiments. Calf-fed Holstein steers supplemented with VM increased (*P* ≤ 0.03) overall average daily gain (ADG), feed efficiency (G:F), observed/expected net energy (NE) values for maintenance and gain, and final body weight (BW). Cattle fed VM also increased (*P* ≤ 0.04) carcass weight, dressing percent, and longissimus muscle area. However, there was no effect (*P* ≥ 0.22) of VM supplementation on any other carcass characteristics. Calf-fed Holstein steers fed 100% MP requirements during the initial 112-d feeding period had greater (*P* ≤ 0.02) ADG, G:F, observed/expected NE values for maintenance and gain, and live BW compared with steers fed 87% of the expected MP requirements. However, there was no effect (*P* ≥ 0.17) of MP supply during the initial 112-d period on overall (342 d) growth performance measurements. The incidence of liver abscesses was low (averaging 7.7%) and not affected by dietary treatments. We conclude that, independent of MP supplies, supplemental VM enhances overall growth performance and efficiency of energy utilization of calf-fed Holstein steers.

## INTRODUCTION

Calf-fed Holstein steers in the southwestern United States are usually fed diets containing 12% to 13% of crude protein (Zinn et al., 2005; Vasconcelos and Galyean, 2007), with urea as the primary or sole source of supplemental N. Although these diets meet average metabolizable protein (MP) and amino acid requirements for the overall feedlot period (300 to 350 d; NASEM, 2000), they do not meet the amino acid requirements of calf-fed Holstein steers during the first 112 to 168 d of the early growing phase of those animals (Zinn and Shen, 1998; Zinn et al., 2007). Recently, Torrentera et al. (2017) and Montaño et al. (2019) concluded that the addition of rumen-protected amino acids (methionine and lysine) to the diet of growing Holstein calves might enhance gain efficiency and dietary energetics during the early growing phase. According to Loerch et al. (1983) and Stock et al. (1981), blood meal (BM) has above 82% of ruminal undegradable protein. Moreover, Titgemeyer et al. (1989) stated that among four different protein sources in the diet, BM had the greatest amount of lysine reaching the duodenum of beef steers.

Virginiamycin (VM) is an antimicrobial additive that inhibits the growth of Gram-positive bacteria (Cocito, 1979; De Araújo et al., 2016) and the growth of ruminal lactic acid-producing bacteria, thereby reducing the risk of lactic acidosis and associated digestive dysfunctions (Owens et al., 1998). Previous research has also demonstrated that VM is an effective tool to control liver abscess incidence in feedlot cattle (Rogers et al., 1995; Nagaraja and Chengappa, 1998; Nagaraja and Lechtenberg, 2007, Tedeschi and Gorocica-Buenfil, 2018). In an earlier study (Salinas-Chavira et al., 2016), enhancements in gain efficiency and estimated dietary NE were augmented in diets formulated to meet metabolizable amino acid requirements during the initial 112 d on feed.

Therefore, the objective of the present study was to further evaluate the influence of VM supplementation on cattle growth performance and liver abscess incidence, and the effect of feeding 100% vs. 87% of MP requirements during the initial 112 d on growth performance, efficiency of energy utilization, and carcass characteristics of calf-fed Holstein steers.

## MATERIALS AND METHODS

Animal care and handling techniques were approved by the University of California Animal Care and Use Committee (#20548).

### Animal Processing, Housing, and Feeding

One hundred sixty-eight Holstein steer calves (133.4 ± 7.9 kg) were used to evaluate treatment effects on growth performance and dietary net energy (NE). Calves originating from Tulare, California, were shipped to the University of California Desert Research Center, Holtville. Upon arrival, calves were vaccinated for infectious bovine rhinotracheitis (IBR) virus, bovine virus diarrhea (BVD) virus Types 1 and 2, parainfluenza3 (PI_3_) virus and bovine respiratory syncytial virus (BRSV) (Bovi-shield Gold One Shot, Zoetis Animal Health, New York, NY), and clostridial infections (UltraBac 7, Zoetis Animal Health, New York, NY); treated for parasites (Dectomax Injectable, Zoetis Animal Health, New York, NY); and injected with 500,000 IU vitamin A (Vital EA-D, Stuart Products, Amarillo, TX). Steers were balanced by weight and assigned to 28 pens (6 steers/pen). During the initial 112-d feeding period, dietary treatments consisted of two levels of MP (100% vs. 87% of expected requirements; NASEM, 2000) supplemented with or without 22.5 mg/kg VM (Phibro Animal Health, Teaneck, NJ 07666) in a 2 × 2 factorial arrangement (Table 1). The low MP diet (87% of the requirements) is typical of conventional feedlot formulations, utilizing urea as the primary source of supplemental nitrogen. Subsequently (day 112 to 308), supplemental BM was withdrawn. Diets were prepared at weekly intervals and stored in plywood boxes located in the front of each pen. Steers were allowed ad libitum access to water and dietary treatments. Fresh feed was provided twice daily. On days 112 and 224, all steers were injected subcutaneously with 500,000 IU vitamin A (Vital E-A + D, Stuart Products, Bedford, TX) and implanted with Revalor-S (Intervet, Millsboro, DE).

**Table 1.** Composition of experimental diets (DM basis)

	0 VM‖		22.5 mg VM	
Item	Conventional$	MP¶	Conventional	MP
Ingredient composition, % DM				
Sorghum Sudan	8.00	8.00	8.00	8.00
Alfalfa hay	4.00	4.00	4.00	4.00
Tallow	2.50	2.50	2.50	2.50
Molasses, cane	4.00	4.00	4.00	4.00
Dry distillers grains plus solubles	10.00	7.00	10.00	7.00
Porcine blood meal	0.00	3.00	0.00	3.00
Steam-flaked corn	68.09	68.09	68.06	68.06
Urea	1.15	1.15	1.15	1.15
Limestone	1.68	1.68	1.68	1.68
Dicalcium phosphate	0.10	0.10	0.10	0.10
Magnesium oxide	0.15	0.15	0.15	0.15
V_max_	0.00	0.00	0.03	0.03
TM salt†	0.30	0.30	0.30	0.30
Nutrient composition,‡ DM basis				
Dry matter, %	87.90	87.90	87.90	87.90
NE_m_, Mcal/kg	2.21	2.19	2.21	2.19
NEg, Mcal/kg	1.54	1.52	1.54	1.52
Crude protein, %	14.30	16.20	14.30	16.20
Rumen DIP, %	63.80	57.30	63.80	57.30
Rumen UIP, %	36.20	42.70	36.20	42.70
Ether extract, %	6.70	6.40	6.70	6.40
Ash, %	5.78	5.71	5.78	5.71
Nonstructural CHO, %	58.00	57.50	58.00	57.50
NDF, %	17.70	16.30	17.70	16.30
Calcium, %	0.80	0.81	0.80	0.81
Phosphorus, %	0.35	0.34	0.35	0.34
Potassium, %	0.77	0.75	0.77	0.75
Magnesium, %	0.28	0.27	0.28	0.27

CHO, carbohydrates; DIP, degradable intake protein; NDF, neutral detergent fiber; UIP, undegradable intake protein.

Trace mineral salt contained: CoSO_4_, 0.068%; CuSO_4_, 1.04%; FeSO_4_, 3.57%; ZnO, 0.75%; MnSO_4_, 1.07%; KI, 0.052%; and NaCl, 93.4%.

Based on tabular values for individual feed ingredients (NASEM, 2000).

VM: V-Max, Phibro Animal Health, Ridgefield Park, NJ.

Conventional: Diet not supplemented with BM providing 87% of expected MP requirements during the initial 112-d feeding period (NASEM, 2000).

MP: Diet supplemented with 3% BM providing 100% of expected MP requirements during the initial 112-d feeding period (NASEM, 2000).

### Carcass Data

All harvest data were obtained from the Carcass Data Service team of West Texas A&M Beef Carcass Research Center (Canyon, TX 79016). Hot carcass weights (HCW) and liver scaring and abscess measures were obtained at the time of slaughter. After carcasses chilled for 24 h, the following measurements were obtained: Longissimus muscle (LM) area (cm^2^) by direct grid reading of the muscle at the 12th rib; subcutaneous fat (cm) over the LM at the 12th rib taken at a location 3/4 the lateral length from the chine bone end (adjusted by eye for unusual fat distribution); estimated kidney; and marbling score (USDA, 1997; using 3.0 as minimum slight, 4.0 as minimum small, 5.0 as minimum modest, and 6.0 as minimum moderate).

### Estimation of Dietary NE

Energy gain (EG, Mcal/d) was calculated by the equation: $EG=0.0557W^{0.75}\times ADG^{1.097}$, where EG is the daily deposited energy, and W is the body weight (BW; NASEM, 1984). Maintenance energy (EM, Mcal/d) was calculated by the equation: $EM=0.086{W^{0.}}^{75}$ (Garrett, 1971; Fox and Black, 1984; NASEM, 1988). From the derived estimates of energy required for maintenance and gain, the net energy for maintenance (NE_m_) and net energy for gain (NE_g_) values of the diet were obtained using the quadratic formula: $x=(-b\;-\surd b^{2}-4ac)/2c$, where a=−0.41EM, b=0.877EM+0.41DMI+EG, and c=−0.877 DMI, and NEg=0.877 NEm−0.41 (Zinn and Shen, 1998). In the determination of ADG, interim and final weights were reduced by 4% to account for digestive tract fill. The final shrunk weight was carcass adjusted by dividing HCW by the decimal fraction of the average dressing percentage of all steers in the study (0.621).

### Statistical Design and Analysis

Data for growth performance variables were analyzed in a randomized complete block design, with a 2 × 2 factorial arrangement of treatments, considering initial shrunk weight groupings for blocks, and pen as an experimental unit, according to the following statistical model: $Y_{ij}=\mu +B_{i}+T_{j}+\varepsilon_{ij}$, where μ is the common experimental effect, B_i_ represents initial weight block effect, T_j_ represents dietary treatment effect, and ε_ij_ represents the residual error (Statistix 10, Analytical Software, Tallahassee, FL). Treatment main effects and interactions were tested by means of orthogonal contrasts.

## RESULTS AND DISCUSSION

There were no VM × MP supplementation interactions (*P* ≥ 0.14). Therefore, detailed discussions relative to the interaction will not be addressed.

### Virginiamycin

Treatment effects on cattle growth performance and dietary NE values are presented in Table 2. Supplementation with 22.5 mg/kg VM increased (*P* ≤ 0.03) the overall ADG, G:F, observed/expected NE values for maintenance and gain, and final BW. Overall, ADG (1.44 kg/d) was in close agreement with the projected value (1.43 kg/d; Torrentera et al., 2016). Similarly, overall dietary NE estimated from growth performance measures agreed (98%) with expected NE values estimated based on tabular feed values from NASEM (2000).

**Table 2.** Treatment effects on growth performance and dietary NE energetics of feedlot steers

	0 VM‖		22.5 mg VM		SEM	*P*-value	
Item	Conventional$	MP¶	Conventional	MP		MP	VM
Days on test	342	342	342	342			
Pen replicated	7	7	7	7			
Live weight, kg†							
Initial	131.3	131.2	131.9	131.4	0.37	0.43	0.23
112 d	275.7	284.2	279.4	284.7	2.3	<0.01	0.36
224 d	453.3	456.2	458.3	459.7	3.7	0.57	0.27
Final	616.5	613.3	629.9	631.4	6.9	0.90	0.03
ADG, kg							
1 to 112 d	1.29	1.37	1.32	1.37	0.02	<0.01	0.46
112 to 224 d	1.59	1.54	1.59	1.56	0.03	0.17	0.51
224 to 342 d	1.38	1.33	1.45	1.45	0.03	0.46	0.01
1 to 342 d	1.42	1.41	1.46	1.46	0.02	0.94	0.04
DMI, kg/d							
1 to 112 d	5.80	5.93	5.78	5.88	0.06	0.08	0.58
112 to 224 d	8.42	8.34	8.25	8.35	0.11	0.96	0.48
224 to 342 d	10.40	10.30	10.40	10.20	0.11	0.17	0.5
1 to 342 d	8.26	8.23	8.18	8.18	0.08	0.85	0.41
G:F							
1 to 112 d	0.22	0.23	0.23	0.23	0.002	0.02	0.13
112 to 224 d	0.19	0.18	0.19	0.19	0.003	0.13	0.23
224 to 342 d	0.13	0.13	0.14	0.14	0.003	0.89	<0.01
1 to 342 d	0.17	0.17	0.18	0.18	0.002	0.97	<0.01
Dietary ME, Mcal/kg‡							
Maintenance							
1 to 112 d	1.84	1.89	1.88	1.91	0.02	0.05	0.10
112 to 224 d	2.18	2.18	2.25	2.21	0.03	0.53	0.11
224 to 342 d	2.15	2.13	2.24	2.28	0.03	0.77	<0.01
1 to 342 d	2.12	2.11	2.19	2.19	0.02	0.98	<0.01
Gain							
1 to 112 d	1.21	1.25	1.24	1.26	0.01	0.05	0.10
112 to 224 d	1.50	1.50	1.56	1.53	0.02	0.53	0.11
224 to 342 d	1.48	1.46	1.56	1.59	0.03	0.77	<0.01
1 to 342 d	1.45	1.44	1.51	1.52	0.02	0.98	<0.01
Observed/ expected dietary NE							
Maintenance							
1 to 112 d	0.84	0.86	0.85	0.87	0.01	<0.01	0.10
112 to 224 d	0.99	0.99	1.02	1.00	0.01	0.53	0.11
224 to 342 d	0.98	0.97	1.02	1.04	0.01	0.77	<0.01
1 to 342 d	0.96	0.96	0.99	1.00	0.01	0.86	<0.01
Gain							
1 to 112 d	0.79	0.83	0.81	0.84	0.01	<0.01	0.10
112 to 224 d	0.99	0.99	1.02	1.00	0.02	0.53	0.11
224 to 342 d	0.97	0.96	1.02	1.05	0.02	0.77	<0.01
1 to 342 d	0.95	0.95	0.99	1.00	0.01	0.86	<0.01

DMI, dry matter intake.

Live weight reduced by 4% to account for gut fill.

Calculated based on the cattle performance according to Zinn and Shen (1998).

VM: V-Max, Phibro Animal Health, Ridgefield Park, NJ.

Conventional: Diet not supplemented with BM providing 87% of expected MP requirements during the initial 112-d feeding period (NASEM, 2000).

MP: Diet supplemented with 3% BM providing 100% of expected MP requirements during the initial 112-d feeding period (NASEM, 2000).

Similar to the current study, Salinas-Chavira et al. (2016) observed that VM supplementation increased gain efficiency (G:F) and dietary NE when calf-fed Holstein steers were supplemented with 22.5 mg/kg of VM for 308 d. However, Latack et al. (2021) reported no effect of VM supplementation on calf-fed Holstein steers feedlot growth performance when VM was fed at 16 mg/kg of dry matter (DM) for 321 d. In a summary review study by Rogers et al. (1995), the authors reported that maximal growth performance response to VM in traditional beef breeds was observed at levels of supplementation of 19.3 to 27.3 mg/kg of diet DM. Therefore, based on the current study and previous research from our group (Salinas-Chavira et al., 2009, 2016; Latack et al., 2021), supplementation of growing-finishing diet to calf-fed Holstein steers with VM enhances cattle growth performance in the feedlot when supplemented at 22.5 mg/kg of DM.

Compared with non-supplemented calves, supplementation with 22.5 mg/kg of DM of VM increased (*P* ≤ 0.04) carcass weight, dressing percentage, and LM area (Table 3). These enhancements were consistent with the increase in final BW (Table 2). Consistent with previous studies involving calf-fed Holstein steers (Salinas-Chavira et al., 2009, 2016; Latack et al., 2021), the influence of supplemental VM on kidney, pelvic, and heart (KPH), fat thickness, and marbling score was not appreciable (*P* ≥ 0.22).

**Table 3.** Treatment effects on carcass characteristics and health status of calf-fed Holstein steers

	0 VM‖		22.5 mg VM			*P*-value	
Item	Conventional$	MP¶	Conventional	MP	SEM	MP	VM
Carcass wt, kg	383.1	381.2	391.5	392.4	4.3	0.9	0.03
DP	61.9	61.7	62.4	62.6	0.32	0.93	0.04
KPH fat, %†	2.42	2.35	2.41	2.37	0.05	0.33	0.91
Fat thickness, cm	0.93	0.88	0.86	0.92	0.06	0.93	0.84
LM area, cm2	74.9	76	76.9	82.5	1.8	0.08	0.03
Marbling score‡	4.64	4.29	4.25	4.45	0.23	0.76	0.62
Abscessed liver, %	9.52	11.9	9.52	0	3.8	0.36	0.14
Morbidity, %	4.76	4.76	2.38	0	3.3	0.72	0.29
Sick days	0.17	0.24	0.17	0	0.16	0.78	0.48

DP, dressing percentage.

KPH fat as a percentage of carcass weight.

Coded: minimum slight = 3, minimum small = 4, minimum modest = 5, minimum moderate = 6, and so on.

VM: V-Max, Phibro Animal Health, Ridgefield Park, NJ.

Conventional: Diet not supplemented with BM providing 87% of expected MP requirements during the initial 112-d feeding period (NASEM, 2000).

MP: Diet supplemented with 3% BM providing 100% of expected MP requirements during the initial 112-d feeding period (NASEM, 2000).

Although VM supplementation has been reported as an effective method to control the incidence of liver abscess in feedlot cattle (Rogers et al., 1995; Nagaraja and Lechtenberg, 2007, Tedeschi and Gorocica-Buenfil, 2018), there was no effect (*P* = 0.14) of VM supplementation on the percentage of abscessed livers (Table 3). However, the incidence of liver abscesses was low (averaging 7.7%) compared with values of 30% or greater previously reported in the literature (Rogers et al., 1995; Tedeschi and Gorocica-Buenfil, 2018). Rogers et al. (1995) and Tedeschi and Gorocica-Buenfil (2018) noted that feedlot cattle management practices influence the relative effectiveness of VM. Supplemental VM did not affect (*P* = 0.29) calf morbidity, which was likewise low, averaging 3%.

### Metabolizable Protein

Calf-fed Holstein steers fed 100% of the expected MP requirements during the initial 112-d feeding period (NASEM, 2000) had greater (*P* ≤ 0.02) ADG, G:F, observed/expected NE values for maintenance and gain, and live BW at 112 d compared with steers fed 87% of the expected MP requirements (Table 2). Previous research has reported that as MP supply increased, cattle feedlot growth performance also increased, reaching a plateau when supplies of limiting amino acids are met (Zinn and Owens, 1993; Zinn et al., 2007). Similar to the current study, Zinn et al. (2007) and Salinas-Chavira et al. (2016) observed that meeting MP requirements enhanced cattle growth performance during the initial growing phase of calf-fed Holstein steers compared with conventional steam-flaked corn-based diet with urea as the sole source of supplemental N. Although cattle in the 100% MP group had greater growth performance during the first 112 d compared with the control group, observed NE values for cattle fed a diet meeting MP requirement were still less than expected based on NASEM (2000; 86.5% for maintenance and 83.5% for gain) during the initial 112-d period.

Zinn et al. (2007) and Salinas-Chavira et al. (2016) observed that when calf-fed Holstein steers were supplemented with 100% MP requirements during the growing phase, the enhancement in growth performance during that period remained appreciable at the time of harvest (more than 300 d on feed). In contrast, in the present study, there was no effect (*P* ≥ 0.17) of early supply of MP on the overall (342 d) cattle growth performance (Table 2). The basis for this effect is not certain but may reflect the somewhat less-than-expected enhancement in gain efficiency as well as diluting effect of the protracted overall days on feed.

Moreover, in order to meet 100% of MP requirements of growing calf-fed Holstein steers during the initial 112 d on feed, porcine BM was supplemented at 3% of the diet (DM basis), replacing dry distillers’ grains plus solubles. Previous research has reported that BM supplementation enhances the amount of ruminal undegradable protein compared with other protein sources, such as soybean meal and canola meal (Loerch et al., 1983; Titgemeyer et al., 1989; Piepenbrink and Schingoethe, 1998). However, in its drying process, BM undergoes varying degrees of nonenzymatic browning (e.g., Maillard reactions), thereby decreasing the amount of amino acids (particularly lysine) available for absorption in the small intestine. According to Titgemeyer et al. (1989), BM had the largest amount of nonavailable nitrogen for either ruminal degradation or small intestine absorption when compared with other protein sources. With NASEM (2000), it is assumed that 80% of dietary protein that escapes ruminal degradation is digested in the small intestine. Consistent with Titgemeyer et al. (1989), it appears that this may not have been the case in the present study and may explain the less-than-expected enhancement in growth performance and observed/expected dietary energy during the initial 112-d period.

Supplemental MP requirements during the initial 112-d period did not affect (*P* ≥ 0.33) dressing percentage, KPH, fat thickness, marbling score, or incidence of liver abscess. However, there was a trend (*P *= 0.08) for greater (5.6%) LM area of cattle supplemented to meet 100% of the expected MP requirements during the initial 112-d feeding period (Table 3). Likewise, Zinn et al. (2000) and Zinn et al. (2007) observed that enhancing the growth performance of calf-fed Holstein steers during the initial growing phase increased carcass LM area. In contrast, Salinas-Chavira et al. (2016) observed that although supplementation to meet MP requirements enhanced initial 112-d and overall 308-d growth performance, it did not appreciably affect carcass characteristics.

## CONCLUSIONS

Supplementation of a steam-flaked corn-based growing-finishing diet with 22.5 mg/kg VM enhanced the overall growth performance and efficiency of energy utilization of calf-fed Holstein steers during a 342-d feeding period. Supplementing to meet MP requirements during the initial 112 d on feed enhanced cattle growth performance and efficiency of energy utilization during that initial period. However, the long-term benefit of that response was not appreciable.
